# Filamin-A Regulates Neutrophil Uropod Retraction through RhoA during Chemotaxis

**DOI:** 10.1371/journal.pone.0079009

**Published:** 2013-10-25

**Authors:** Chunxiang Sun, Carol Forster, Fumihiko Nakamura, Michael Glogauer

**Affiliations:** 1 The Faculty of Dentistry, University of Toronto, Toronto, Ontario, Canada; 2 Translational Medicine Division, Department of Medicine, Brigham and Women's Hospital, Harvard Medical School, Boston, Massachusetts, United States of America; National Jewish Health and University of Colorado School of Medicine, United States of America

## Abstract

Filamin-A (FLNa) has been shown to be a key cross-linker of actin filaments in the leading edge of a motile melanoma cell line, however its role in neutrophils undergoing chemotaxis is unknown. Using a murine transgenic model in which FLNa is selectively deleted in granulocytes, we report that, while neutrophils lacking FLNa show normal polarization and pseudopod extension, they exhibit obvious defects in uropod retraction. This uropod retraction defect was found to be a direct result of reduced FLNa mediated activation of the small GTPase RhoA and myosin mediated actin contraction in the FLNa null cells. This results in a neutrophil recruitment defect in FLNa null mice. The compensatory increase in FLNb levels that was observed in the FLNa null neutrophils may be sufficient to compensate for the lack of FLNa at the leading edge allowing for normal polarization, however this compensation is unable to regulate RhoA activated tail retraction at the rear of the cell.

## Introduction

Cell migration is a fundamental process in a diverse array of physiologic processes, including organism development, wound healing, and immune function. This central cellular event involves a progression through a repeating set of coordinated steps; extension of the leading edge (lamellopodium), attachment of the leading edge to the substratum, forward flow of the cytosol, and retraction of the rear of the cell (uropod). In order for coordinated forward cell movement to occur, these steps must be carried out in an orderly, sequential and repeating manner. Identifying the various elements regulating these various steps is critical to the understanding of cell migration. 

The neutrophil is an excellent model system for studying cell migration and chemotaxis, since these highly motile cells display a very well-defined polarity during chemotaxis that allows for the visualization of the fluid transitions between the steps of cell migration. Initially, using fibroblasts and now neutrophils, it has been shown that members of the Rho GTPase family including Rac, cdc42, and RhoA play crucial roles during cell migration [1,2] 

While Cdc42 is responsible for filopodia formation and cell polarity, Rac localizes to, and is necessary for, efficient actin polymerization and protrusion of the leading edge [2,3]. Rho on the other hand, localizes to the rear of the migrating cell and is necessary for myosin-based contraction of the cell’s uropod or trailing edge [1,3,4].

Actin binding proteins regulate cell migration through activation of actin filament assembly and disassembly. They are also key regulators of the actin cytoskeletal architecture/morphology. Filamin family members are critical regulators of the actin cytoskeletal architecture [5] . These large cytoplasmic proteins cross link actin filaments into orthogonal three-dimensional networks in the actin cortex. The three human isoforms; Filamin A (FLNa), Filamin B (FLNb) and Filamin C (FLNc) share 70% sequence homology, although they have unique tissue expression with both FLNa and FLNb being ubiquitously expressed, while FLNc shows more restricted expression limited to skeletal and cardiac muscles during adult life. 

The prototype and first identified filamin, FLNa, was discovered in monocyte/macrophages and shown to be a potent cross linker of actin filaments in the cell cortex [6] . Through its ability to crosslink actin filaments into highly branched (orthangonal) networks, it is able to stabilize actin filaments in the leading edge of migrating cells thereby facilitating efficient cell migration [5-7]. Previous studies investigating FLNa’s role in cell migration have been primarily carried out in human melanoma cells lacking the FLNa protein. These FLNa null cells display severe impairments in the earliest stages of cell locomotion; namely cell polarization and pseudopod extension which manifests as continuous circumferential blebbing of the plasma membrane [8]. However, more recently, FLNa has also been shown to be an important integrator of signals in a large variety of cellular processes through its binding to many signaling proteins including the Rho family of small GTPases[ 5,9]. 

RhoA, along with the other members of the Rho GTPases, functions as a molecular switch, cycling between an inactive GDP-bound state and an active GTP bound state. Their state of activity is regulated by the opposing effects of guanine nucleotide exchange factors (GEFs), which enhance the GTP bound form, and the GTPase-activating proteins (GAPs), which increase the intrinsic rate of hydrolysis of bound GTP [2,10,11]. We and others have previously shown that Rho activity is required in the uropod in order to carryout coordinated uropod retraction during chemotaxis[12]. In this study, we demonstrate that FLNa plays an essential role in the activation of the RhoA small GTPase and myosin based uropod contraction during neutrophil chemotaxis.

## Materials and Methods

### *FLNa* Mutant Mice

All procedures were carried out in accordance with the Guide for the Humane Use and Care of Laboratory Animals, and were approved by the University of Toronto Animal Care Committee.

Mice containing the conditional knock out of the X-linked *FLNa* gene were generated as described previously [13]. Briefly, a conditional knockout strategy was used with loxp sites inserted into introns 2 and 7 of the mouse FLNa gene (*FLNa* ^*c/c*^ or male *FLNa* ^*c/y*^ mice). In order to target deletion of the *FLNa* gene to neutrophils these mice were bred with mice expressing cre recombinase under control of the LysM promoter which is granulocyte specific [14]. The *LysM* gene encodes lysozyme, an enzyme that is expressed at high levels in granulocyte precursor cells. Cre-mediated recombination deletes exons 3-7, producing a non-sense mutation with early FLNa truncation at amino acid 121. We have previously demonstrated that this results in >90% deletion of the floxed gene in neutrophils [15-17]. 

### PCR Genotyping Analyses of Mice

**Figure 1:**
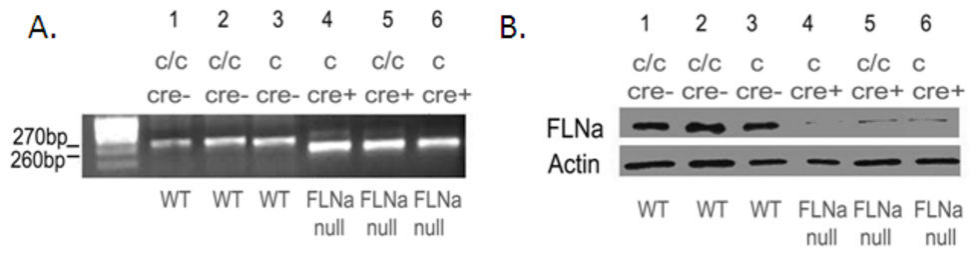
Genotyping and FLNa Expression (**A**) PCR confirmation of FLNa in peritoneal neutrophils. Primer mFNhe5 and mFBam3 yielded products of sizes 270 bp representing the wild-type gene product (lanes 1, 2 and 3), while mFNhe5 and Mf1Ne8R yielded products of sizes of 260 bp representing the FLNa null gene product (lanes 4, 5 and 6). Note a small residual band in the cre recombinase expressing mice. (**B**) Representative immunoblot of FLNa expression in peritoneal neutrophils. As expected from PCR genotyping mice were generated with neutrophils lacking FLNa expression designated as FLNa null (lanes 4, 5 and 6). Note the presence of a faint residual band of FLNa in lanes 4, 5 and 6 which suggests that the LysM Cre mouse incompletely eliminates the flanked loxP DNA from all neutrophils. Antibody against β-actin was used as a loading control. c/c denotes that the FLNa gene is floxed and conditional. Since the FLNa gene is on the X chromosome males are denoted with a single c.

Tail snips were used to prepare DNA for PCR analysis [18]. PCR genotyping was performed by simultaneous amplification of the wild-type (*FLNa* ^*+*^), conditional (*FLNa* ^c^), and null (*FLNa* ^-^) alleles using four primers: mFNhe5, TCTTCCTCTTTCAGCTGG; mFBam3, ACAACTGCTGCTCCAGAG; Syn 1, CTGCATTACCGGTCGATGCA; Syn 2,ACGTCCACCGGCATCAACGT. mFNhe5 and mFBam3 were included in a single PCR and yielded products of sizes 270 bp (*FLNa* ^+^) and 260 bp (*FLNa* ^-^), which were analyzed on 3% agarose gels (Figure 1A). The presence of the *LysMcre* allele was assessed by a PCR using primers: Syn 1and Syn 2 which yielded a product of size 300 bp, described previously [14].

### FLNa and FLNb Analysis

Isolated bone marrow neutrophils (40,000 PMNs for each genotype) were exposed to fMLP (10^-6^M) (Sigma, St. Louis, MO) at 37°C for the 2 minutes, immediately lysed with laemmli buffer, boiled for 10 minutes, and subjected to 7.5% SDS-PAGE and immunoblotted with either Anti-FLNa or Anti-FLNb[19]. 1:100 with 5% BSA Tris-buffered saline-Tween (TBS-T) at 4°C overnight. Membranes were washed 3 times for 10 minutes with TBS-T followed by incubation with goat anti-mouse IgG peroxidase conjugates (Amersham Pharmacia Biotech, Buckinghamshire, England). Densitometry analysis of the Antigen-antibody complexes was carried out. Two standard curves were created with known amounts of the FLNa (5, 10, 20, 40 ng) and FLNb protein (2ng, 4ng, 6ng, 8ng) as a means to determine the total protein concentrations in wild-type and FLNa null neutrophils.

### Neutrophil preparations

Mice were killed by CO_2_ inhalation. Femurs and tibias were removed and bone marrow was isolated. Bone marrow cells were layered onto discontinuous Percoll (Sigma, Oakville, ON, Canada) gradients of 82%/65%/55%. Mature neutrophils were recovered at the 82%/65% interface and were positive for Gr-1 and Mac-1 as shown by flow cytometry. More than 85% of the cells isolated were neutrophils, as assessed by Wright-Giemsa staining [20]. Viability, as determined by trypan blue exclusion, was more than 90%.

### Chemotaxis Assay

Bone marrow neutrophils were suspended in HBSS and 1% gelatin. A neutrophil suspension (1 x 10^6^/mL) was allowed to attach to bovine serum albumin (BSA) (1mg/ml)-coated glass coverslips (22 x 40 mm) at 37°C for 10 minutes. The coverslip was inverted into a Zigmond chamber, and 100 µL HBSS media was added to the right chamber with 100 µL HBSS media containing fMLP[10^-6^M] added to the left chamber. Time-lapse video microscopy was used to examine neutrophil movements in Zigmond chambers. The microscope (Nikon Eclipse E400) was equipped with differential interference contrast optics and X 40 objective. Images were captured at 20-second intervals with Nikon Eclipse E1000 Microscope. Cell-tracking software (Retrac version 2.1.01 Freeware) was used to characterize cellular chemotaxis from the captured images. Image J software was used to measure the length of cells. Data came from three independent experiments.

### Chemokinesis Assay

The procedure was the same as the chemotaxis assay but 100 µL HBSS media containing fMLP[10^-6^M] was added to the right and left chambers. Data of the mean value came from three independent experiments.

### Neutrophil polarization without fMLP

A neutrophils suspension in HBSS (1x 10^6^/ml ) were allowed to attach on BSA(1 mg/ml) coated cover glasses for 15 minutes; images were captured with Nikon Eclipse E1000 Microscope. Three independent experiments were performed.

### In vivo chemotaxis and peritonitis model

1 ml (2 mM) sodium periodate was injected into murine peritoneal cavities. After 3 hours, mice were euthanized with CO_2_. PBS media (5 ml) was injected into the murine peritoneal cavities and the lavage fluid was recollected. The fluid was centrifuged at 150*g* for 5 min at 4 °C and cell pellets were resuspended in PBS. The total number of cell was counted with Beckman Coulter. Cell monolayers from the peritoneal cavity prepared using Cytospin 2 (Shandon Southern Products, Astmoor, UK). Cell morphology was determined on the slides stained with Diff-Quick (Fisher Scientific, Pittsburgh, PA). Three independent experiments were carried out.

### GST-Pull down

The Pak-binding domain (PBD) and Rhotekin-binding domain (RBD) assays were carried out as described previously [21]. Briefly, isolated bone marrow neutrophils were exposed to fMLP at 37°C for the indicated period of time. They were immediately lysed, and glutathione-S-transferase (GST)-PBD or GST-RBD beads (Amersham Pharmacia Biotech Buckinghamshire, England) was added to the lysates. The GST-PBD or GST-RBD were recovered with Sepharose beads and immunoblotting was completed using either a Cdc42 antibody (B-8; Santa Cruz Biotechnology), Rac1 antibody (Upstate Cell Signaling Solutions (Charlottesville, VA), or a RhoA antibody (sc-418; Santa Cruz Biotechnology). Image J software version 1.31v (National Institutes of Health) was used for blot densitometry. Data was normalized to resting wild-type neutrophils and are expressed as the mean value from 3 separate experiments.

### Phospho-myosin light chain 2 Assay

Isolated bone marrow neutrophils were exposed to fMLP (10^-6^M) at 37°C for 120 seconds, immediately lysed with laemmli buffer, boiled for 10 minutes, and subjected to 15% SDS-PAGE. Membranes were blocked and immunoblotted with Phospho-Myosin Light Chain 2 (Ser19) antibody (Cell Signaling Technology; Beverly, MA) 1:1000 with 5% BSA Tris-buffered saline-Tween (TBS-T) at 4°C overnight. Membranes were washed 3 times for 10 minutes with TBS-T followed by incubation with goat anti-Rabbit IgG peroxidase conjugates (Amersham Pharmacia Biotech, Buckinghamshire, England). Antigen-antibody complexes were visualized on X-ray film by enhanced chemiluminescence (ECL; Perkin Elmer Life Sciences, Boston, MA). Data is from four separate experiments.

### Phospho-Myosin Light Chain 2, Rho A & FLNa Immunostaining

One million neutrophils were attached on BSA-coated slides for 10 min at 37°C and stimulated with fMLP for 2 minutes followed by fixation with 4% PFA. Fixed cells were washed in PBS, permeabilized with 0.1% Triton X-100 in PBS for 4 minutes, and blocked with 1% BSA for 30 minutes. Cells were incubated with anti- Phospho-Myosin Light Chain 2, RhoA (Cell Signaling Technology) or FLNa, diluted 1:100, and detected with Alexa Flour 488 goat anti–rabbit IgG (Invitrogen). Cells were also stained with 1:400 rhodamine phalloidin at room temperature for 30 min followed by epifluorescence microscopy analysis. Images were visualized using an Eclipse E100 (Nikon), a 40/0.95 Plan Apo objective (Nikon), and a digital camera (C4742-80; Hamamatsu). Images were acquired using Simple PCI software version 5.3 (Compix). Three independent experiments were carried out.

### Cell Adhesion Assay

Aliquotes of 5x10^5^ neutrophils were plated into a 96 wells plate coated with BSA (100mg/ml), fibronectin(1 mg/ml, 10µg/ml) or fibrinogen( 50µg/ml) for 30 minutes. The total number of cells was counted under microscope before turning over the plate and spinning at 33 g for 30 seconds. The wells were rinsed with PBS and the total number of cells was counted again. The percentage of adherent cells before and after spinning was determined. Data was from three independent experiments.

### Statistical Analysis

Data are presented as the mean ± SD. Statistical comparisons were made by a two-tailed t-test. In cases with multiple comparisons ANOVA with Tukey for post-hoc testing was carried out. Blots and micrographs are representative of at least three separate experiments. Differences with P values ≤ 0.05 were considered statistically significant.

## Results

### Generation of mice with neutrophils lacking FLNa expression

**Figure 2:**
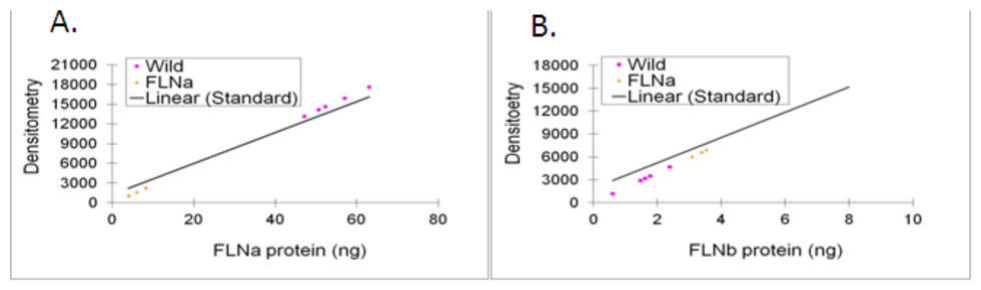
Standard Curve of FLNa and FLNb (**A**) FLNa and (**B**) FLNb standard curves were created by loading known amount of FLNa (5, 10, 20, 40ng) and FLNb ( 2, 4, 6, 8ng) in to 7.5% SDA-PAGE gels along with 40 thousand neutrophils from WT (Yellow) and FLNa null (Pink) mice. Densitometry of the blots was correlated with the amount of protein. (N=3-5). (**C**) The concentration of FLNa and FLNb in wild type and FLNa null neutrophils. The change in each protein between WT and Null cells is noted.

To directly study the specific roles of Filamin-A (FLNa) during neutrophil chemotaxis, we bred FLNa conditional mice with mice expressing cre recombinase downstream of the graunulocyte specific LysM promoter. Previous studies have shown that the *LysM* ^*cre*^ allele leads to deletion efficiencies of 83–99% in mature granulocytes of floxed alleles [14]. PCR analysis of genomic DNA from peritoneal neutrophils from FLNa null (females: *FLNa* ^*c*/-^ *LysM* ^cre/+^ or males: *FLNa* ^*c*/y^ *LysM* ^cre/+^) mice demonstrated near complete conversion of the conditional allele into the null allele (Figure 1A). A faint wild type band is noted in the Cre-recombinase samples. Western blot analysis of protein from pertitoneal neutrophils from FLNa null mice also demonstrates a faint residual FLNa band following incubation with a FLNa antibody (Figure 1B). This is likely due to a combination of minor cell contamination from peritoneal preparations and a small percentage of cells that did not undergo recombinase mediated deletion of the *FLNa* gene [14]. In order to assess FLNa protein expression in the null cells, Western blot analysis using rFLNa as a standard demonstrated that ~40, 000 wild-type neutrophils contain (53.97 ± 2.76 ng ) of FLNa and the same number of FLNa null (*FLNa* ^*c*/-^ *LysM* ^cre/+^) neutrophils showed 6.09 ng ± 0.80 ng of FLNa (Figure 2 A, Table 1). Since recombination mediated by Cre recombinase either occurs or does not occur, the neutrophils will either have no FLNa expression or will have normal FLNa expression levels. As described previously and shown here (Figure 1A), The LysM Cre mouse eliminates the flanked loxP DNA in at least 88% of neutrophils [14]. It is likely higher as the residual FLNa protein detected in the Western blots results from a combination of non-neutrophil cell contamination as our isolation procedure has ~5% contamination of other cell types suggesting that only a small fraction (~7%) of neutrophils retain FLNa expression. 

**Table 1:** 

**Protein (ng/cell)**	**WT**	**FLNa null**	**Percent Change from WT to FLNa**
**FLNa**	1.35 x10^-3^±1.54x10^-4^	1.52 x10^-4^±5.28x10^-5^	-89%
**FLNb**	9.60x10^-6^±4.03x10^-6^	2.09 x10^-5^±1.43x10^-6^	+54%

### Upregulation of Filamin-B does not compensate for FLNa deficiencies

To determine if the FLNb isoform was compensating for the loss of FLNa, the levels of FLNb expression were measured in the control and FLNa null cells. Protein levels from Western blots of the wild-type and FLNa-null neutrophils were applied to the standard curve that was created using known amounts of FLNb antigen to determine the total amount of FLNb protein present in the neutrophils. In the absence of the FLNa protein expression, neutrophils show a significant 2-fold increase in FLNb protein expression compared to wild-type neutrophils (Figure 2B,Table 1). 

### FLNa is required for tail retraction

**Figure 3:**
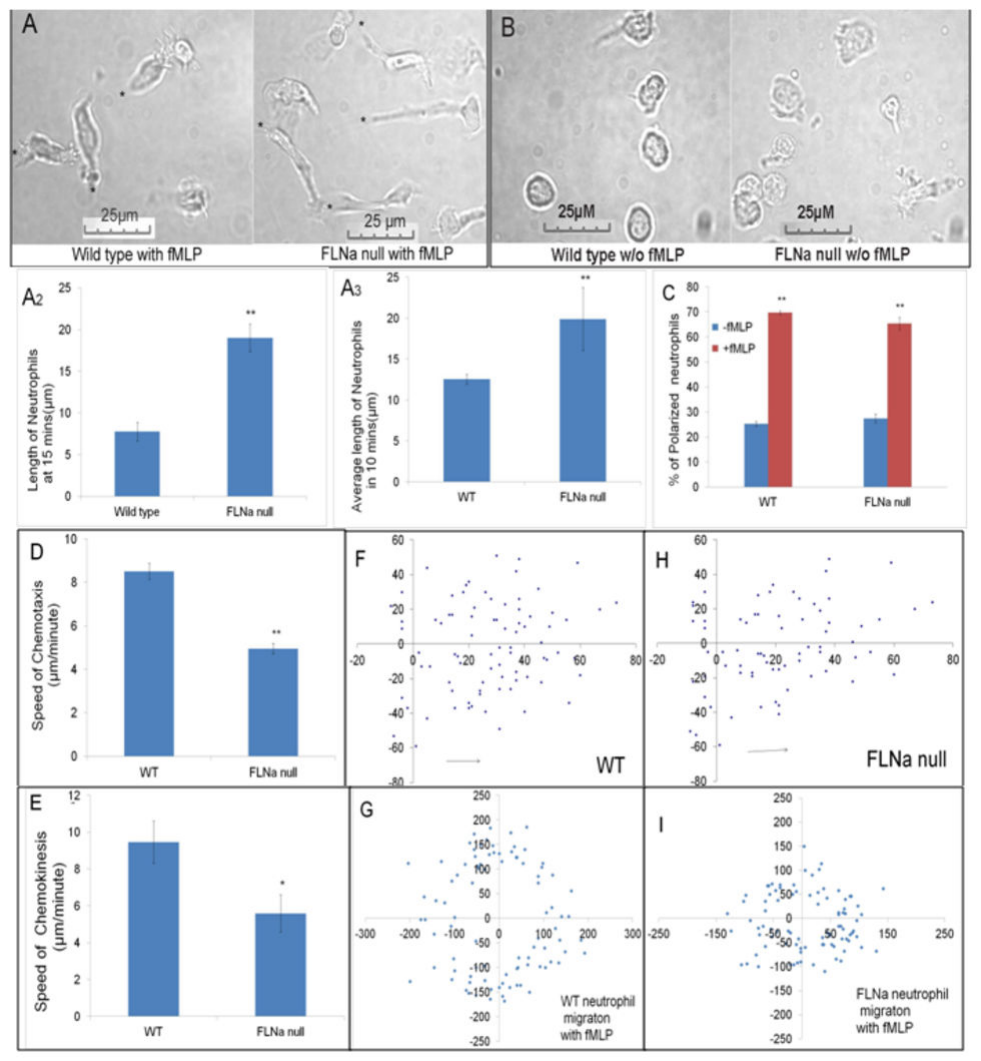
FLNa alters cell translocation but not polarization (A) WT and FLNa null neutrophils show similar pseudopod extension in the fMLP gradient, however the FLNa cells show poor tail retraction. (* trailing uropod). (**A2**) The mean length of neutrophils after 15 minutes of fMLP. (**A3**) The average length of neutrophils during10 minutes of fMLP stimulation. n= 90 cells (**B**) Lack of cell polarization without fMLP after 20 minutes. (**C**) FLNa null cells show no deficiency in fMLP mediated polarization. (**D**, **F**, **H**) Chemotaxis: FLNa null cells migrate toward fMLP (towards right) slower than WT cells (**E**, **G**, **I**) Chemokinesis: FLNa null cells show reduced speed/translocation during chemotaxis compared to WT cells. Bar diagrams represent the average of 3 independent experiments ± SD. *P < .05 ** *P* < .01.

**Figure 4:**
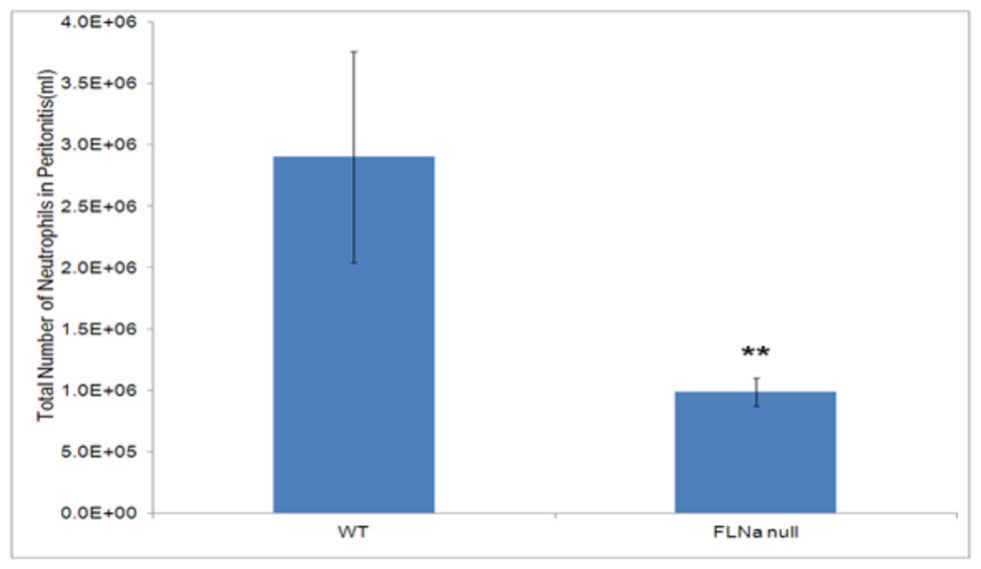
FLNa required for recruitment to peritoneum (A) Neutrophil Peritoneal Recruitment: FLNa null mice display reduced neutrophil peritonitis induced recruitment.

Previous studies with FLNa-deficient melanoma cells show that FLNa is required for cell polarization and migration toward a chemoattractant [8]. We observed that neutrophils deficient in FLNa, are able to polarize and undergo chemotaxis in response to an fMLP stimulus in a similar manner to WT neutrophils (Figure 3A, B, C). A Zigmond chamber chemotaxis assay was used to measure both chemokinesis (100nm) and chemotaxis. These experiments revealed that FLNa null neutrophils demonstrate significantly diminished chemokinesis speeds compared with wild-type neutrophils (Figure 3 E, G, I). FLNa null neutrophils also displayed significantly reduced speeds compared with WT neutrophils in the chemotactic gradient, although the moderate amount of migration that did occur in the FLNa null cells resulted in translocation toward the chemoattractant source (Figure 3 D, F, H). The reduced speed for FLNa null neutrophils was maintained over 30 minutes of migration toward fMLP (data not shown). To confirm that this phenotype was maintained in vivo, a peritonitis model showed that FLNa deficient neutrophils displayed reduced accumulation during an acute inflammatory response (Figure 4). To characterize the tail retraction defect, we measured the average neutrophil length after 15 minutes and the average change in neutrophil length over 10 minutes. Both analyses showed that the FLNa null cells displayed a mean length more than double the WT neutrophils (Figure 3A2, A3). To rule out the possibility of lysMCre being involved in the neutrophil phenotype, in vivo and in vitro chemotaxis assays showed no difference between lysMCre lacking and expressing neutrophils. (data not shown).

### FLNa regulates neutrophil RhoA activity in the uropod

**Figure 5:**
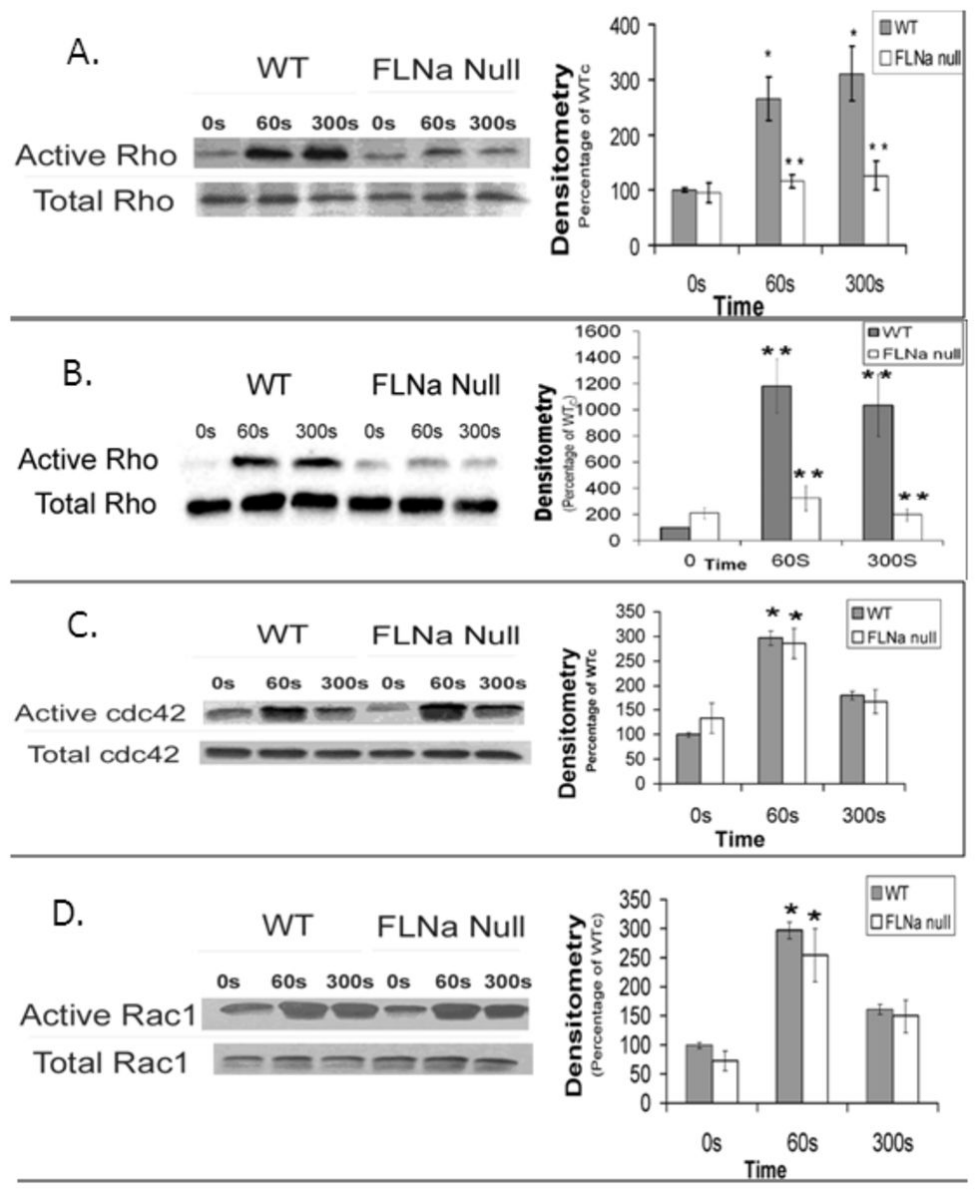
Rho A activity is reduced in FLNa null cells WT and FLNa null neutrophils in suspension (**A**) or plated on dishes (**B**) for 20 minutes are stimulated with 1μM fMLP for 30, 60, and 300 seconds. GTP-RhoA is detected by immunoblot assay. Immunoblot and densitometry analysis shows a decrease of active RhoA in FLNa null neutrophils over a 300 sec time-frame compared to wild-type cells. (**C**) cdc42 and (**D**) Rac1 activities in FLNa null cells: FLNa null cells show no defect in cdc42 and Rac1 activation as seen in Rho. Bar diagrams represent the average of 3 independent experiments ± SD. ** *P* < 0.01.

**Figure 6:**
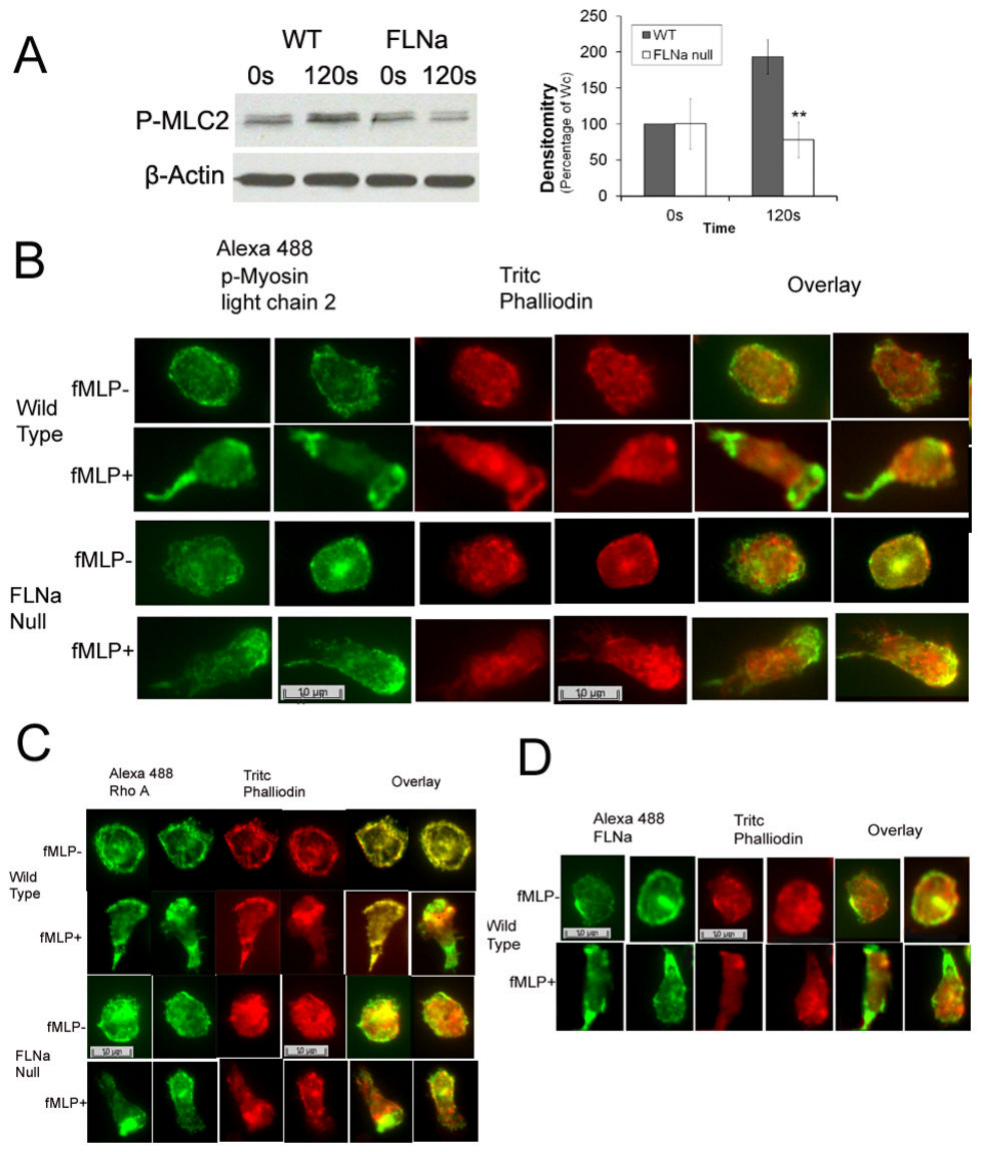
FLNa regulates localization of p-Myosin Light Chain-2 (p-MLC2) and Rho A in the uropod (A) Reduced p-MLC2 levels in FLNa null neutrophils. Immunoblot analysis shows a significant reduction in p-MLC2 in FLNa null neutrophils following 120sec of fMLP (10^-6^) compared to WT levels. Mean ± SD of four mice per group. * significance of p< .05 within a group and (**) significance of p<.05 between groups (B) p-MLC2 is absent in the tails of FLNa null neutrophils after stimulation with fMLP at 120s. C) Rho A is absent in the tails of FLNa null neutrophils following fMLP stimulation at120s. (D) FLNa primarily localizes to the tail of wild type neutrophils after fMLP stimulation at 120s.

Since FLNa null neutrophils exhibited poor tail retraction, we assessed the role of FLNa in the activation of the small RhoA small GTPase following fMLP (10^-6^M) stimulation. Western blot analyses of total cellular extracts showed that the levels of active RhoA was significantly reduced in both attached FLNa null neutrophils (by 83% at 60 sec and 300 sec of fMLP stimulation) and those in suspension (by 53% at 60 sec and 59% at 300 sec of fMLP stimulation) when compared to wild-type controls (Figure 5A, B). Conversely, the activation levels of the small GTPases, Rac1 and cdc42, were undisturbed in FLNa null neutrophils compared to wild-type cells (Figure 5C, D). To further verify FLNa regulation of Rho A in the uropod, subcellular localization of FLNa and Rho A were investigated. Fluorescence microscopy images displayed the abundant FLNa and Rho A at the uropod in WT cells, but an absence of Rho A in the uropod in FLNa deficient cells (Figure 6C, D).

### Filamin-A regulates Myosin II–mediated contractility through Rho in the uropod

In order to determine whether the FLNa-dependent activation of RhoA could account for the observed tail retraction defect, we determined the localization and activation state of myosin light chain 2 using an antibody to phosphorylated (Ser19) myosin regulatory light chain 2. Myosin has been previously shown to function in uropod retraction through its role in regulating actin filament contraction [22,23]. In murine neutrophils, we observed that cells without FLNa displayed a significant inhibition of normal fMLP-mediated increase in myosin light chain 2 phosphorylation in the neutrophils (Figure 6A). Overall levels of myosin light chain 2 phosphorylated at Ser19 were decreased by more than 84% in FLNa null cells, while the overall activation of myosin, as measured by myosin light chain 2 phosphorylation at Ser19, was significantly increased in fMLP stimulated wild-type neutrophils. Immunolocalization of phospho- myosin light chain 2 in fMLP-activated mouse neutrophils demonstrated localization to the uropod in WT cells, but absence in the uropod of FLNa-null cells (Figure 6B).

### Neutrophil-substrate adhesiveness depend on FLNa

**Figure 7:**
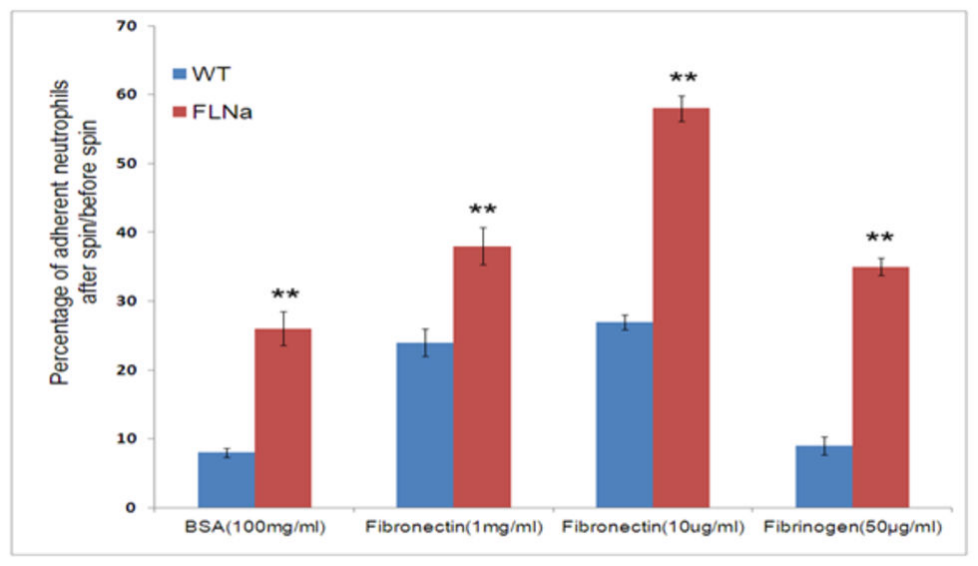
FLNa regulates neutrophil-substrate adhesiveness FLNa deficiency increases neutrophil-substrate adhesion. The number of cells was counted prior to plate inversion and spinning at33 RCF. The wells are rinsed with PBS and the total number of cells counted again. The percentage of cells attached after spinning were calculated. Three independent experiments were carried out. Error bars are ± SD. **p< .01.

To investigate the role FLNa in neutrophil-substrate adhesion, we compared the percentage of cells remaining attached after a shear force applied to 96-well plates that were coated with different integrin ligands. The results showed that FLNa deficient cells had a significant increase in adhesiveness to substrates coated with BSA, fibronectin and fibrinogen (Figure 7). 

## Discussion

FLNa was first identified as the prototypic actin binding protein. However in recent years it has been recognized that this important actin binding and filament cross-linking protein has numerous binding sites for signaling proteins and may play important roles in a number of signal transduction cascades [5,24]. In this study, we investigated the role of FLNa in neutrophil chemotaxis. Using primary neutrophils derived from a murine transgenic conditional FLNa knock-out mouse, we show that FLNa regulates cell locomotion efficiency through its role in activation of RhoA-myosin mediated uropod retraction. 

### FLNa and chemotaxis

Previous studies looking into FLNa’s role in directed cell migration (chemotaxis) have been carried out primarily in a human melanoma cell line lacking FLNa. These experiments supported the role of FLNa in the early phases of cell migration. These melanoma cells have been shown to be incapable of polarization and locomotion due to their inability to regulate and stabilize their cortical actin cytoskeleton which manifests as continuous circumferential surface blebbing [8]. Neutrophils are a commonly used cell model for studying chemotaxis, due to the distinct cell polarization that results in a well-defined leading edge and a characteristic tail or uropod. We have shown here that neutrophils lacking FLNa are able to polarize normally and migrate up a chemoattractant gradient; however, they are unable to retract their uropod resulting in an elongated cell body during chemotaxis. This data appears to be inconsistent with previous melanoma cell work in this area and suggests that FLNa is not essential at the leading edge of migrating neutrophils. 

This inconsistency can be explained by the possibility that in FLNa null neutrophils, FLNb may be partially capable of compensating for the lack of FLNa. In particular, this may be true at the front of the cell, where FLNa is essential for actin crosslinking during polarization and leading edge extension. This explanation is not unreasonable, as several other cell lines including fibroblasts, vascular endothelial cells and neural crest cells exhibit normal filamentous actin (F-Actin) structure, motility, and locomotion in the absence of FLNa, leading the authors to question FLNa’s role in F-actin polymerization and cross-linking while also not ruling out the possibility that FLNb and FLNc may compensate for FLNa to maintain motility in these cells [13,25,26]. It is important to note that despite the observed increase in FLNb levels, the total amount of this protein present in the FLNa null cells is small, on the order of 66X’s less, in comparison to the levels of FLNa normally present in neutrophils. However, it may be possible, that FLNb is sufficiently concentrated at the leading of the cell to replace FLNa levels there but unable to replace FLNa in the uropod to activate RhoA during chemotaxis. Unfortunately an antibody for FLNb immunolocalization is not available to test this hypothesis.

### FLNa and RhoA

Neutrophil chemotaxis is dependent on the establishment and maintenance of cell polarity. A well-defined leading edge and uropod requires the localization and activation of specific Rho GTPases. Unlike Cdc42 and Rac which localize to and are responsible for the formation and protrusion of the leading edge, RhoA localizes to and is responsible for regulating the myosin II-based contraction and retraction of the trailing uropod [27,28]. Our data shows RhoA is in both the leading edge and uropod in WT cells but only in the leading edge of the FLNa null cells. While Pertz et al. [29] observed that RhoA is primarily found in the leading edge of fibroblasts, previous work from our lab has shown that RhoA does also localize to the uropod of neutrophils [12] and is required for normal cell migration.

In muscle and non-muscle cells, the activation of Myosin-II is primarily through the phosphorylation of the myosin light chain at serine 19 [22,29]. The actin based myosin motor at the rear of the cell is essential for tail retraction and can be activated through two separate pathways. The first requires intracellular calcium, which binds calmodulin and activates myosin light chain kinase, which then directly phosphorylates and activates the myosin binding subunit of the myosin regulatory light chain [30]. The second also involves the myosin light chain, which can be activated through the small GTPase RhoA pathway. When RhoA is active in the rear of the cell, it phosphorylates and activates Rho-kinase (ROCK), which can either directly phosphorylate and activate the myosin binding subunit of myosin light chain or can phosphorylate and inactivate myosin phosphatase, preventing myosin phosphatase from dephosphorylating and inactivating the myosin light chain. In addition to the localization and activation of RhoA and MLC in the uropod, a neutrophil must also break the newly formed adhesive contacts with the substratum at the rear of the cell to allow the actinomyosin motor to retract the tail and maintain persistent forward motility. Myosin light chain kinase-inhibited neutrophils show a similar tail retraction defect to the FLNa null cells, where a thin region of non-retractable membrane at the uropod remains tightly anchored to the substratum and often became elongated as the cell extended a new leading edge forward [31]. These studies illustrate that FLNa mediates RhoA activation in chemotaxing neutrophils. Potential Rho regulatory mechanisms to explore involve RhoA GEFs and GAPs including RhoGEF Lbc or TRIO. In addition to manipulating the activation state of RhoA, these two proteins have been shown to interact with FLNa, making them worthwhile candidates to explore further.
